# Variation, Sex, and Social Cooperation: Molecular Population Genetics of the Social Amoeba *Dictyostelium discoideum*

**DOI:** 10.1371/journal.pgen.1001013

**Published:** 2010-07-01

**Authors:** Jonathan M. Flowers, Si I. Li, Angela Stathos, Gerda Saxer, Elizabeth A. Ostrowski, David C. Queller, Joan E. Strassmann, Michael D. Purugganan

**Affiliations:** 1 Department of Biology and Center for Genomics and Systems Biology, New York University, New York, New York, United States of America; 2 Department of Ecology and Evolutionary Biology, Rice University, Houston, Texas, United States of America; Fred Hutchinson Cancer Research Center, United States of America

## Abstract

*Dictyostelium discoideum* is a eukaryotic microbial model system for multicellular development, cell–cell signaling, and social behavior. Key models of social evolution require an understanding of genetic relationships between individuals across the genome or possibly at specific genes, but the nature of variation within *D. discoideum* is largely unknown. We re-sequenced 137 gene fragments in wild North American strains of *D. discoideum* and examined the levels and patterns of nucleotide variation in this social microbial species. We observe surprisingly low levels of nucleotide variation in *D. discoideum* across these strains, with a mean nucleotide diversity (π) of 0.08%, and no strong population stratification among North American strains. We also do not find any clear relationship between nucleotide divergence between strains and levels of social dominance and kin discrimination. Kin discrimination experiments, however, show that strains collected from the same location show greater ability to distinguish self from non-self than do strains from different geographic areas. This suggests that a greater ability to recognize self versus non-self may arise among strains that are more likely to encounter each other in nature, which would lead to preferential formation of fruiting bodies with clonemates and may prevent the evolution of cheating behaviors within *D. discoideum* populations. Finally, despite the fact that sex has rarely been observed in this species, we document a rapid decay of linkage disequilibrium between SNPs, the presence of recombinant genotypes among natural strains, and high estimates of the population recombination parameter ρ. The SNP data indicate that recombination is widespread within *D. discoideum* and that sex as a form of social interaction is likely to be an important aspect of the life cycle.

## Author Summary

Theories on the evolution of cooperation sometimes hinge on knowledge of genetic relatedness between individuals. *Dictyostelium discoideum* has been a model for the study of key biological phenomena, including the evolution and ecology of social cooperation, but the nature of genetic variation within this species is largely unknown. We determine the levels and patterns of molecular variation in this social species. We find a preference of genetically identical cells to cooperate with each other in forming fruiting bodies, a phenomenon known as kin discrimination. Kin discrimination, however, does not appear to be correlated with overall DNA divergence of the strains. Instead, familiarity appears to breed contempt, as strains from the same geographic location (which possibly encounter each other) show higher levels of kin discrimination than strains found further apart. We also show that sex, which is rarely observed in the laboratory, appears to be widespread in the wild—an interesting finding given that sex in *D. discoideum* is also associated with cooperation between numerous single cells to feed the developing cannibalistic zygote. The finding that sex may occur more frequently in the wild opens the possibility of conducting controlled laboratory matings and developing *D. discoideum* as a genetic model system.

## Introduction

The origin and maintenance of social cooperation is one of the most intriguing aspects of evolutionary history [1]. The evolution of cooperative interactions underlie some of the major evolutionary transitions, giving rise to phenomena as diverse as multicellularity [2], microbial sociality [3] and the development of animal societies [1]. For cooperation to be favored, the individual providing the assistance must gain fitness either directly (as in mutualism) or indirectly when it helps a relative, and thereby passes on more genes than it could alone [1], [4]. Kin selection theory indicates when altruistic helping should evolve, and predicts a dependence on the genetic relatedness among interactants. Social interactions may thus depend on patterns of genetic diversity either across the genome or at key loci and a molecular population genetic study of nucleotide variation can provide insights into the nature of social dynamics within species.

*Dictyostelium discoideum* has been a key model system for understanding the genetic basis of social behavior as well as multicellular development and cell-cell signaling [5], [6]. *D. discoideum* is a soil amoeba mostly distributed in temperate regions of the Northern hemisphere. The 34 Mb genome has been completely sequenced and is organized into six chromosomes that harbor ∼12,500 protein-coding genes [6]. Moreover, various molecular approaches are available to dissect molecular and cellular functions [5], facilitating a genetic analysis of cooperative behavior.

**Figure 1:**
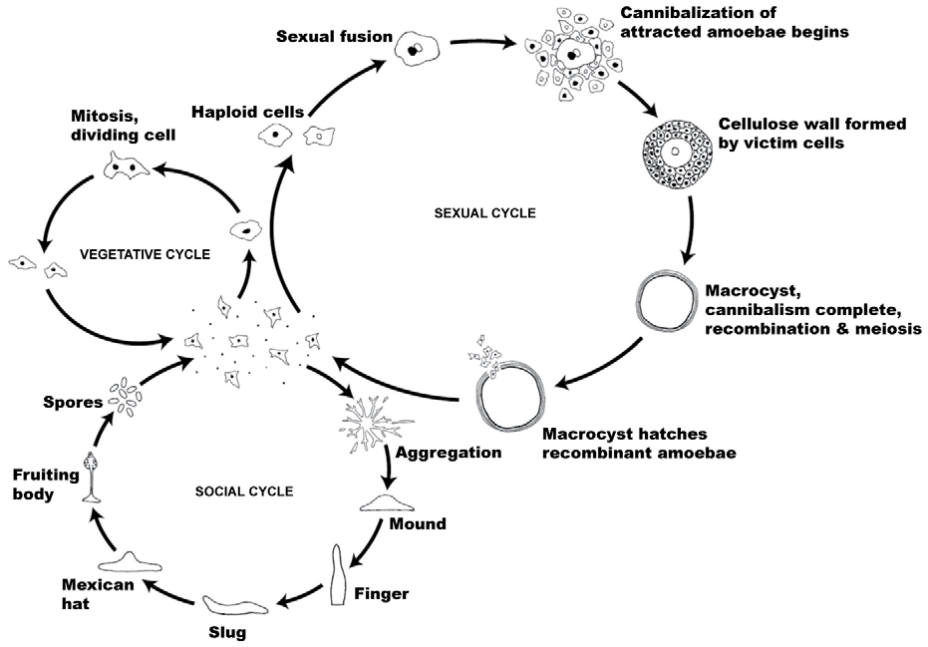
The life cycle of *Dictyostelium discoideum*. Most of its life, this haploid social amoeba undergoes the vegetative cycle, preying upon bacteria in the soil, and periodically dividing mitotically. When food is scarce, either the sexual cycle or the social cycle begins. Under the social cycle, amoebae aggregate and form a motile slug, which ultimately forms a fruiting body. Under the sexual cycle, amoebae aggregate and two cells of opposite mating types fuse, and then begin consuming the other attracted cells. Before they are consumed, some of the prey cells form a cellulose wall around the entire group. When cannibalism is complete, the giant diploid cell or macrocyst eventually undergoes recombination and meiosis.

*D. discoideum* is notable for two social phases in its life cycle, one of which has received much more attention than the other (see Figure 1). A well-studied social phase of the life cycle occurs when starvation conditions lead to a transition from solitary lives as haploid single cells into swarming aggregates that eventually develop into multicellular fruiting bodies composed of haploid spores and stalk cells (see Figure 1) [7]–[12]. Aggregation of individual amoebae is mediated by the chemoattractant cAMP, which triggers the chemotactic movement of cells, the polarized secretion of more cAMP for signal relay, and the initiation of changes in developmental gene expression [5]. Up to 10^5^ individual cells aggregate, leading to the formation of a fruiting body, with the sorus sitting on the top of the stalk. This structure contains cells encapsulated as spores that await dispersal and germination when conditions are favorable for single-cell growth. In this cooperative asexual structure, stalk cells perish and thus exhibit altruistic behavior towards the viable spores which they lift above the substrate where they are more likely to be dispersed [12].

The differentiation of cell fate leads to cooperation as well as competition between individuals from distinct clones and dramatically increases the complexity of this system in the context of social behavior. Although social cooperation in *D. discoideum* strains has been largely studied in cells with the same genetic background, clones with different genetic backgrounds are known to co-occur in the field [13], [14]. It has been demonstrated, for example, that cheating behavior is commonly seen in these chimeric fruiting bodies comprised of disparate strains, in which selfish clones (cheaters) preferentially occupy a larger proportion of spores [12]. Moreover, the degree of kin discrimination has been reported to be positively correlated with genetic distance between strains [15], [16].

The second, little-studied social phase in this species is a sexual phase that represents an alternative albeit enigmatic response to starvation (see Figure 1) [17]–[19]. In the sexual phase, cell fusion from distinct (or sometimes similar) mating types is followed by cAMP-mediated attraction of other solitary cells that are cannibalistically consumed by the diploid zygote through massive phagocytosis. This leads to the formation of macrocysts, which represents an alternative mode of social behavior in *D. discoideum*, since neighboring solitary cells are attracted to the forming zygote, altruistically contribute to the formation of the macrocyst, and are then cannibalized for nutrition (see Figure 1). Being cannibalized may make sense when it benefits a member of ones own clone in the zygote, but it raises interesting potential social conflicts. In a mixture of two clones, the minority member would contribute equally to the zygote, but less than its fair share to the feeding of the zygote. In mixtures of more than two clones, cells unrelated to the zygote may be cannibalized against their interest.

Although the sexual phase is an interesting alternative social mode in *D. discoideum*, its role in the lifecycle of this species remains unclear [17]–[19]. The prevalence of sex in this social amoeba has never been determined, since germination of macrocysts to produce their haploid progeny in the laboratory has been infrequent [17], [20] and there is only one estimate of the zygotic recombination rate [20]. In contrast to other model genetic organisms such as yeast, fruit flies and *C. elegans*, the inability to conduct facile matings between strains in the laboratory has also hampered genetic analysis.

While *D. discoideum* is a key model species in the study of social behavior as well as development and cell-cell-signaling, very little is known about the evolutionary genetics of this organism, including the levels, patterns and distribution of nucleotide variation across the species' range. Understanding the nature of molecular variation in this social microbial species is particularly important given the key role genetic variation can play in the evolution and persistence of social interactions. Moreover, nucleotide variation data can be used to infer the role of sex in the lifecycle of *D. discoideum* in nature, by identifying the extent of recombination in the genome. Here we examine molecular diversity in gene fragments throughout the genome among natural strains collected in the eastern United States, and use these data to infer the molecular population genetics of this species. We find surprisingly low levels of variation in *D. discoideum* and use the distribution of single nucleotide polymorphisms (SNPs) to examine the role that recombination plays in shaping natural genetic diversity in this species. Analysis of the geographic distribution of mating types indicates that different types occur sympatrically, which likely facilitates sexual recombination and the decay of linkage disequilibrium observed in the SNP data. Evidence of recombination and our observation of greater discrimination against non-self during chimeric fruiting body formation in sympatric versus allopatric strains provides new insight into the evolution of cooperation in social microbes.

## Results

### Low level of variation in *D. discoideum*

**Figure 2:**
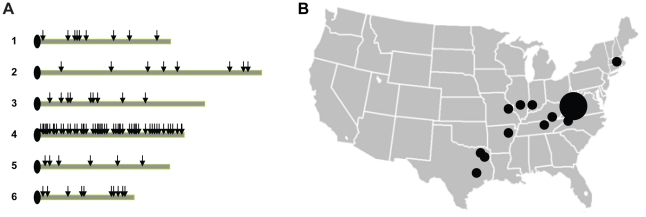
Gene fragments and strain samples used in the study. (A) Approximate locations of gene fragments used in the analysis (depicted by the arrows), with a higher density of fragments sequenced in chromosome 4. (B) Approximate locations of origins of the strains used in the study. The larger circle depicts the greater number of strains collected in sites in Mountain Lake, Virginia.

A total of 137 gene fragments, approximately 400–600 bp in length, from across the *Dictyostelium* genome were chosen for sequencing, with ∼64.6 kb of sequence obtained for each strain. Ninety-four gene fragments are located on chromosome 4, while the remaining 43 are randomly distributed across the rest of the genome. We chose to sample more densely on chromosome 4 to allow for better inference of recombination and linkage disequilibrium (see Figure 2). For this chromosome, gene fragments were spaced at distances between 2.3 and 233.7 kb, with a mean spacing of 55.6 kb.

**Figure 3:**
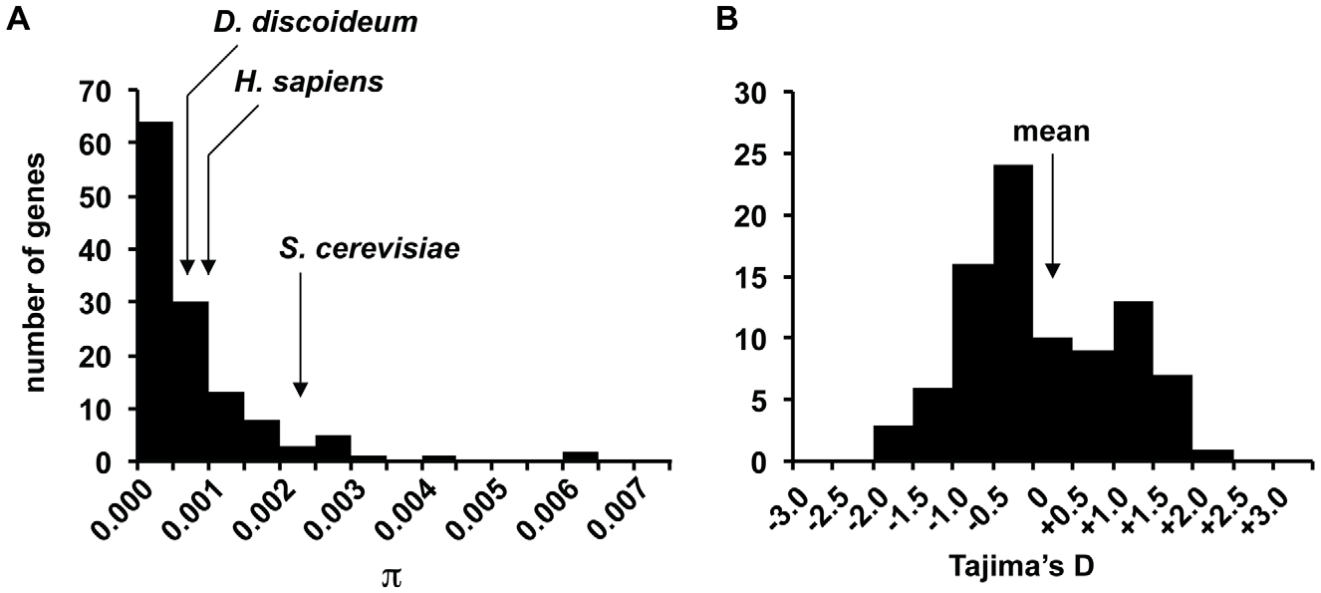
Distribution of nucleotide variation and Tajima's D in gene fragments. (A) The mean π for *D. discoideum* and various other species are indicated. (B) The mean Tajima's D for *D. discoideum* is indicated by the arrow. The smaller number of gene fragments plotted for Tajima's D is due to a large fraction of gene fragments with no variation and whose D estimate could not be determined.

We identified 184 SNPs in the sample, with an average of 1 SNP every ∼350 bps. The level of nucleotide variation is very low with the average number of pairwise differences per site, π, of 0.0008 (see Table 1). One-third of the fragments have no variation among the strains, while the most variable gene fragment had π = 0.0063 (see Figure 3). The level of variation for this haploid species is lower than humans (π = 0.001) and domesticated rice (π = 0.0015), two species that are known to have relatively low levels polymorphism [21], [22].

**Table 1:** Summary population genetic statistics for *D. discoideum*.

Group	n	θ_W_	π	Tajima's D
All	25	0.00076	0.0008	+0.1124
Widespread	13	0.0008	0.0008	+0.0094
Virginia	13	0.00076	0.00072	−0.1032

The low level of variation we observe could arise because half of our sampled isolates come from the Mountain Lake Biological Station, Virginia. We compared levels of variation in these strains (n = 13) to a geographically widespread sample (n = 13); the latter represents all of the non-Virginia strains, a randomly chosen Virginia strain, QS8, and the AX-4 laboratory strain (originally from North Carolina). The mean levels of nucleotide diversity are the same (π = 0.0008) whether we consider all the data or distinguish between the Virginia and geographically widespread samples.

The low level of genome-wide variation is consistent with a recent population bottleneck or possibly a population of small effective size that is at equilibrium between mutation and genetic drift. Recent changes in effective size may be detected with Tajima's D, a population genetic statistic based on the frequency of SNPs that is expected to deviate from zero when populations are not at equilibrium such as following expansion (D<0) or contraction (typically D>0) [23]. Tajima's D estimates for the gene fragments range from −1.5479 to +2.1043, with a mean of +0.1124 (see Figure 3), which is close to the neutral-equilibrium expectation of Tajima's D∼0 and consistent with a population that has not experienced dramatic changes in effective population size in recent history.

### Absence of geographic structure in *D. discoideum* North American strains

The strains used in this study were collected across a wide geographic region in eastern North America, in addition to the Virginia site. A series of clustering analyses were performed to identify genetically similar strains and to identify potential geographic differentiation among strains.

**Figure 4:**
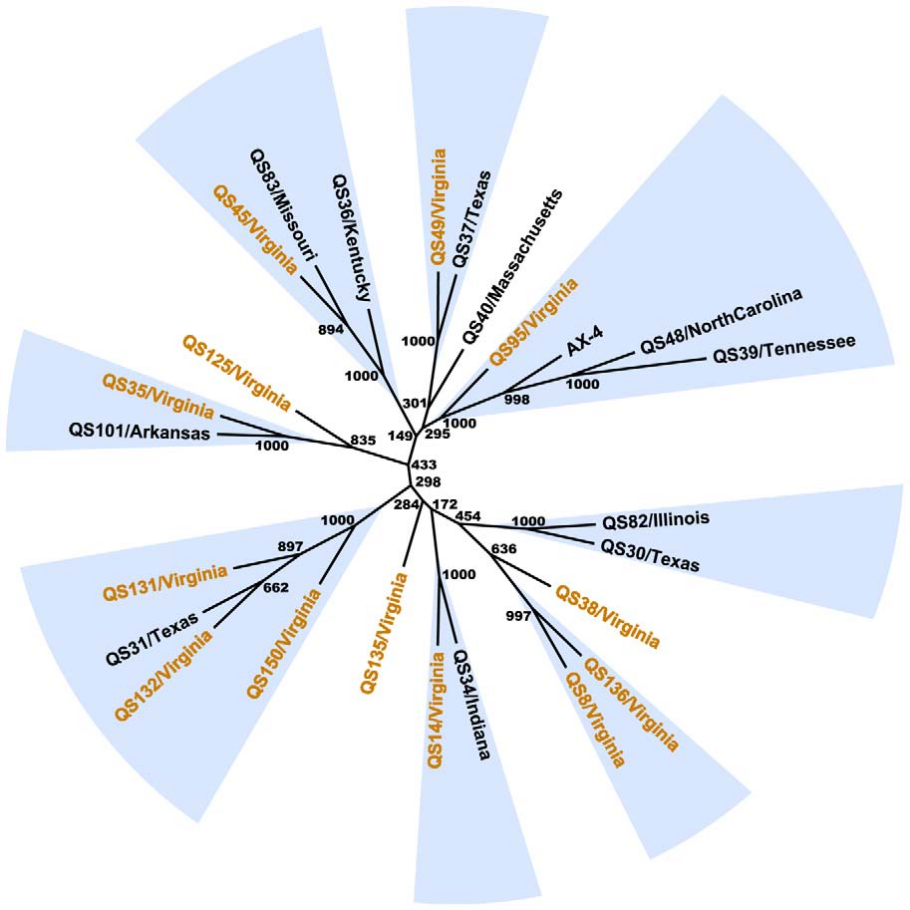
Relationships between *D. discoideum* strains. All STS fragment alignments were concatenated and clustering of strains were estimated with an unrooted neighbor-joining analysis as implemented in PHYLIP, with distances calculated using the Kimura 2-parameter model. Branch bootstrap estimates were obtained from 1,000 replicates. Groups of strains supported by >99% of the bootstrap replicates are highlighted in blue and strains from Virginia are colored in orange.

An unrooted neighbor-joining tree based on concatenated sequenced fragments reveals several clusters of small numbers of strains with strong bootstrap support (see Figure 4). In most cases, each of the supported groups consists of strains from geographically distant locations such as the cluster of QS49 (Virginia) and QS37 (Texas) and a group consisting of QS34 (Indiana) and QS14 (Virginia). Furthermore, the Virginia strains are found throughout the tree with at least one Virginia strain found in all but one of the supported groups. A multiple correspondence analysis (MCA) of SNP variation, which can be considered as a generalization of principal component analysis (PCA) involving categorical (i.e., SNP) data [24] revealed a pattern of strain similarities comparable to the neighbor-joining analysis (see Figure S1). Visualization of the components in a 3-dimensional MCA plot also reveals that the 25 *D. discoideum* strains are generally found in the same clusters (see Figure S1) as observed in the neighbor-joining tree (unpublished observations), although we note that the true genealogical relationships among strains in these analyses may be obscured by recombination.

Finally, a Bayesian clustering analysis based on a population genetic model implemented in the program STRUCTURE suggests that there are at least six and as many as eight clusters (i.e., the change in likelihood, Pr(X|K), begins to plateau at K = 6, and is maximized at K = 8 clusters)[see Figure S2]. However, we observe evidence of admixture among these groups [see Figure S1B], a result consistent with gene exchange (i.e., recombination) among these clusters (see below).

The clustering of strains from geographically distant locations and evidence of extensive admixture in the STRUCTURE analysis indicates no clear geographic differentiation of *D. discoideum* strains in our sample. The mean number of pairwise differences between the Virginia strains is similar to those from the geographically widespread sample (d = 42.732±17.59 in the Virginia sample versus d = 48.61±14.91 in the widespread panel). Moreover, we found no evidence for isolation-by-distance between strains using a Mantel test (r = 0.11, 0.05<P<0.11), and the F_st_ estimate between the Virginia site and the rest of the strains is very low (mean F_st_ = 0.023).

### Relationship among SNP divergence, geographic distance, and fruiting body formation

Kin selection theory predicts that, when cost and benefit involved in a social interaction are fixed, individuals are expected to discriminate based on genetic relatedness and cooperate altruistically more frequently with close relatives. We examined whether kin discrimination is operating in our system by assessing whether strains discriminate in favor of kin (i.e., clonemates) during chimera formation and whether related strains, as measured by sequence similarity, cooperate more than more divergent strains.

We quantified the extent of kin discrimination by estimating relatedness of fruiting bodies relative to sample average (*r_fb_*) in various chimeric mixing experiments. In each experiment, two strains in the single-celled stage were mixed and allowed to form chimeric fruiting bodies with the proportions of each genotype determined in the initial cell mixture and in replicate fruiting bodies (see Materials and Methods). An *r_fb_* = 0 indicates random mixing of cells, while *r_fb_* = 1 is associated with complete kin discrimination favoring fruiting body formation of clonemates. We measured *r_fb_* in mixing experiments between strains of different levels of nucleotide divergence, with SNP differences ranging from 41 to 74 SNPs. We found evidence for between-strain discrimination in chimeric fruiting bodies of *D. discoideum*, with various levels of relatedness in strain mixing experiments, from *r_fb_* = 0.03 to 0.38 with a mean of 0.14±0.03. The strain pair that shows the greatest relatedness is QS49 versus QS150 (both from Virginia).

**Figure 5:**
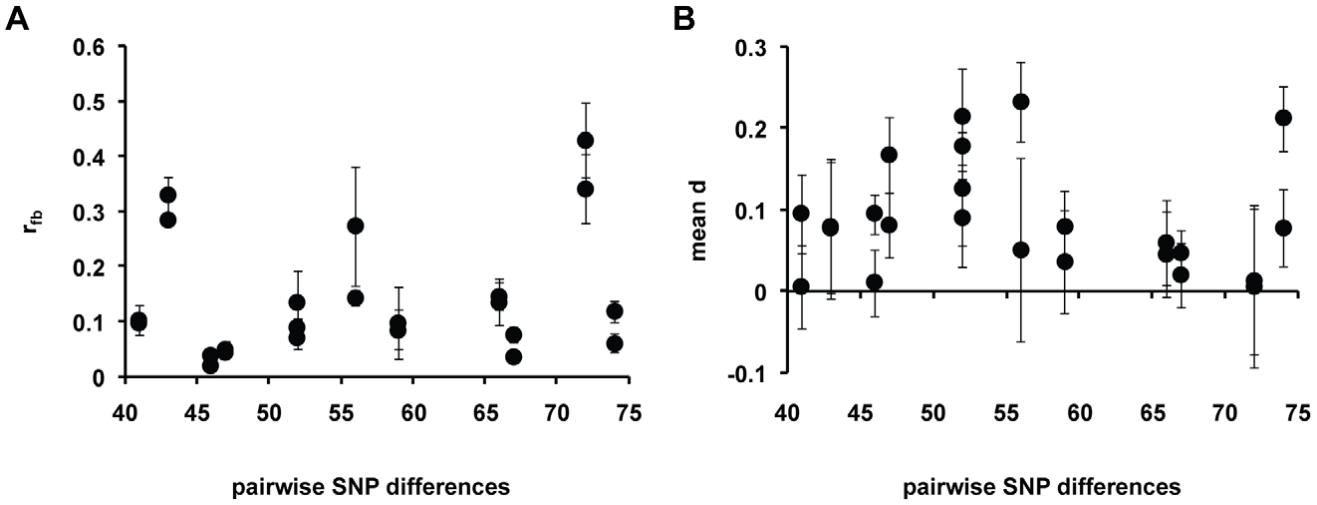
Relationship between social parameters and SNP divergence. (A) Plot of relatedness *r_fb_* versus SNP divergence between strain pair. Mean and standard error of *r_fb_* values were calculated for each SNP used in the strain quantification and shown on the plot. (B) Plot of the means of absolute dominance value *d* versus SNP divergence between strain pairs. Mean and standard error of *d* values were calculated for each SNP used in the strain quantification and shown on the plot.

A plot of the *r_fb_* values among strain pairs of different SNP divergence levels is shown in Figure 5. For this measure, there was no significant correlation with SNP divergence and the fit to the data was low (R^2^ = 0.04). We also employed the Levene's (*LS*) statistic as well as the variance in the arcsine square-root transform [15] of the proportion of strains in the fruiting body as measures of kin discrimination. No correlation between the extent of discrimination and SNP divergence was observed using these two measures (see Figure S3 and Figure S4).

We also examined the dominance of a given participant strain in various chimeric fruiting bodies using the *d* value (see Materials and Methods). A positive *d* value means a specified participant strain occupies a larger proportion in the sorus than would be expected given the strain frequency of the mixed cells in the experiment. We found that dominance occurred between strains in chimeric fruiting bodies; the absolute *d* values range from 0.008 to 0.195 with a mean of 0.087±0.016 among the various chimeric mixing experiments (see Figure 5). The strain pair that shows the greatest dominance is QS132 (Virginia) when in a chimera with QS83 (Missouri); this pair has an absolute *d* of 0.195±0.035. Like *r_fb_*, there is no correlation between mean *d* and the number of SNP differences between strains (R^2^ = 0.019).

One problem in this assay is that the differences in the SNP divergence levels between strain pairs are small and this compromises the resolution of genetic distances. We decided to further compare strain pairs in our experiments by partitioning them into pairs with either low or high divergence. Among all strains in our sample, the median pairwise divergence was 52 SNPs, and we designated strain pairs below and above this median pairwise SNP divergence level as low- and high-divergence pairs, respectively. We found no difference in either kin discrimination or dominance metrics between these two divergence categories (see Table S1).

**Figure 6:**
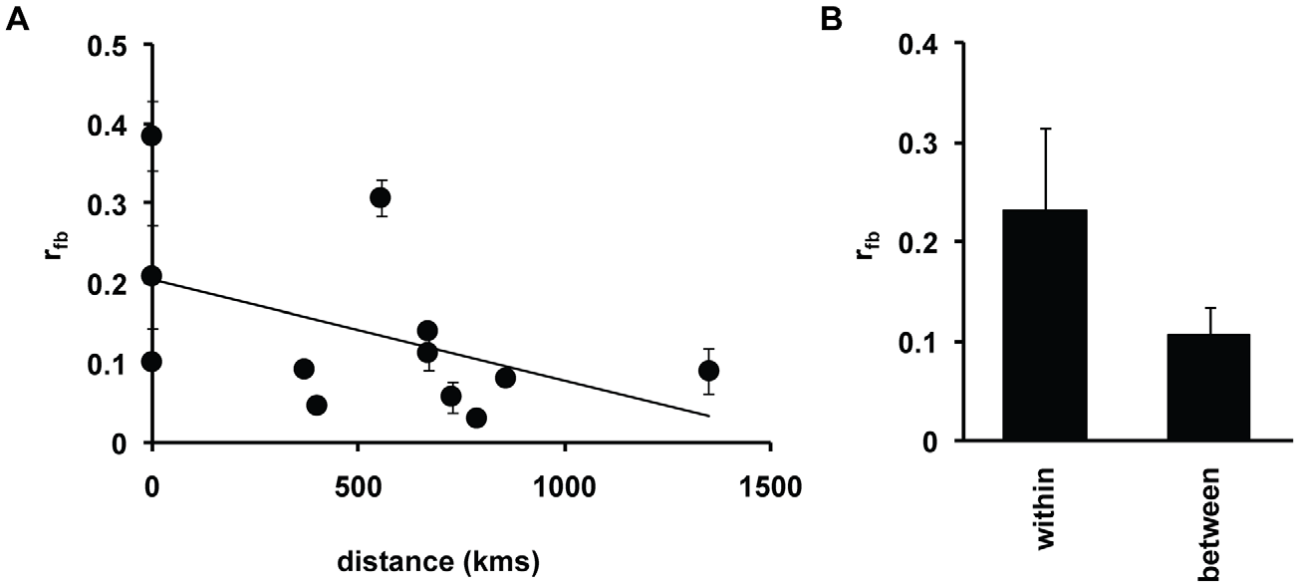
Relationship between kin discrimination and sympatry. (A) The mean relatedness *r_fb_* is plotted against geographic distance of strain locations used in chimeric mixing experiments. Mean and standard error of *r_fb_* values among the three experimental replicates were calculated for each strain pair and shown on the plot. Strains within the Mountain Lake Biological Station site in Virginia are given a distance of 1 km. The line through the data is based on the fit with a linear regression model, and shows a negative relationship of *r_fb_* with geographic distance [R^2^ = 0.22], but this relationship is not significant [P<0.62]. (B) The mean *r_fb_* for strain pairs from the Mountain Lake Biological Station site (left) is compared with those for strain pairs between sites (right)[see Table S5]. Standard errors for the mean *r_fb_* is shown by the bars. The difference in mean *r_fb_* is significant (P<0.038).

Although we did not find a relationship between discrimination and SNP divergence, we observed greater discrimination between strains from the same location versus strains collected from different locations. Using the fruiting body relatedness as our discrimination metric, the level of within-site discrimination is *r_fb_* = 0.23±0.08, while between-site *r_fb_* = 0.12±0.03 (see Figure 6). This increase in fruiting body relatedness in experiments with sympatric strains compared to mixing experiments with allopatric strains is significant (P<0.042). We do not, however, observe a significant increase in dominance in sympatric versus allopatric strains (P<0.49). To examine this further, we also tested for a correlation between kin discrimination, *r_fb_*, and geographic distance. There is a negative relationship between kin discrimination and geographic distance (r^2^ = 0.22), but this correlation is not statistically significant (see Figure 6).

### Rapid decay of linkage disequilibrium and evidence of recombination

The sexual phase in *D. discoideum* has been difficult to observe, and it is possible that sex is rare in this social amoeba. If sex were indeed rare, then we would expect low levels of recombination and high levels of linkage disequilibrium across the genome.

First, we assayed for mating types of our natural strains to determine their geographic distribution. Mating types were assigned by observing macrocyst formation when incubated against tester strains of known mating types A1 and A2. Strains that mate with both testers are designated as mating type A3. Of our 24 tested strains, we were able to assign mating types to 18 strains, with 6 failing to form macrocysts with either tester strain A1 or A2 (see Table 2). We also found that all three mating types were present in the Virginia site, which would indicate that individual amoeba can readily find mating partners within localized areas and thus undertake sexual reproduction.

**Table 2:** Mating types of *D. discoideum* strains.

Strain	Location	Mating Type
QS31	Texas – Houston Arboretum	matA1
QS40	Massachusetts – Mt. Greylock	matA2
QS48	North Carolina – Linville Falls	matA2
QS101	Arkansas-Forest City	matA1
QS30	Texas - Carthage	matA3
QS82	Illinois - Effingham	matA3
QS83	Missouri – St. Louis	unknown
QS38	Virginia – Mt. Lake Biological Station	unknown
QS37	Texas - Linden	unknown
QS34	Indiana - Bloomington	matA2
QS36	Kentucky – Land between the Lakes	unknown
QS39	Tennessee – Indian Gap	unknown
QS8	Virginia – Mt. Lake Biological Station	matA2
QS45	Virginia – Mt. Lake Biological Station	matA3
QS49	Virginia – Mt. Lake Biological Station	matA2
QS35	Virginia – Mt. Lake Biological Station	matA3
QS125	Virginia – Mt. Lake Biological Station	matA3
QS135	Virginia – Mt. Lake Biological Station	matA1
QS14	Virginia – Mt. Lake Biological Station	matA2
QS95	Virginia – Mt. Lake Biological Station	matA2
QS131	Virginia – Mt. Lake Biological Station	unknown
QS150	Virginia – Mt. Lake Biological Station	matA3
QS132	Virginia – Mt. Lake Biological Station	matA1
QS136	Virginia – Mt. Lake Biological Station	matA2

The extent to which sex and recombination occurs in *D. discoideum* can be ascertained by examining the population genetic data for evidence of recombination and measuring the extent of linkage disequilibrium and its rate of decay. We thus characterized linkage disequilibrium among SNPs on chromosome 4, which was more densely sampled for gene fragment re-sequencing than the other chromosomes.

**Figure 7:**
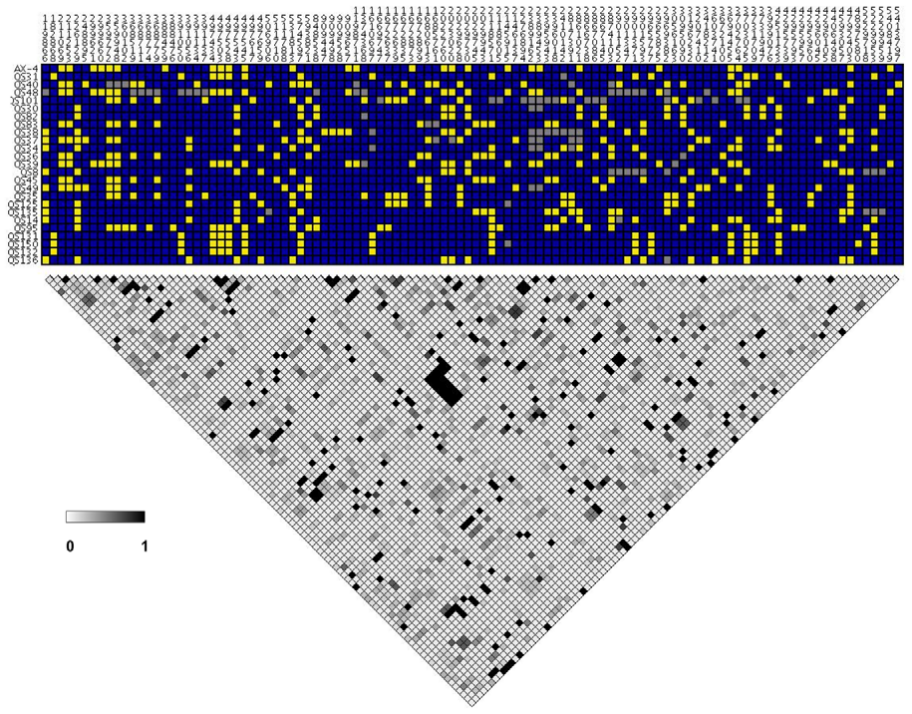
Pairwise linkage disequilibrium in chromosome 4. The segregating SNPs for each strain are shown on top, going from the proximal to distal positions along the chromosome from left to right. Each horizontal row on top is a separate strain. The blue and yellow indicate the major and minor SNP allele, based on frequency in the strains. The gray is missing SNP data. The heat map at the bottom is based on the linkage disequilibrium measure r^2^ between SNPs, with the grayscale legend for the level shown.

**Figure 8:**
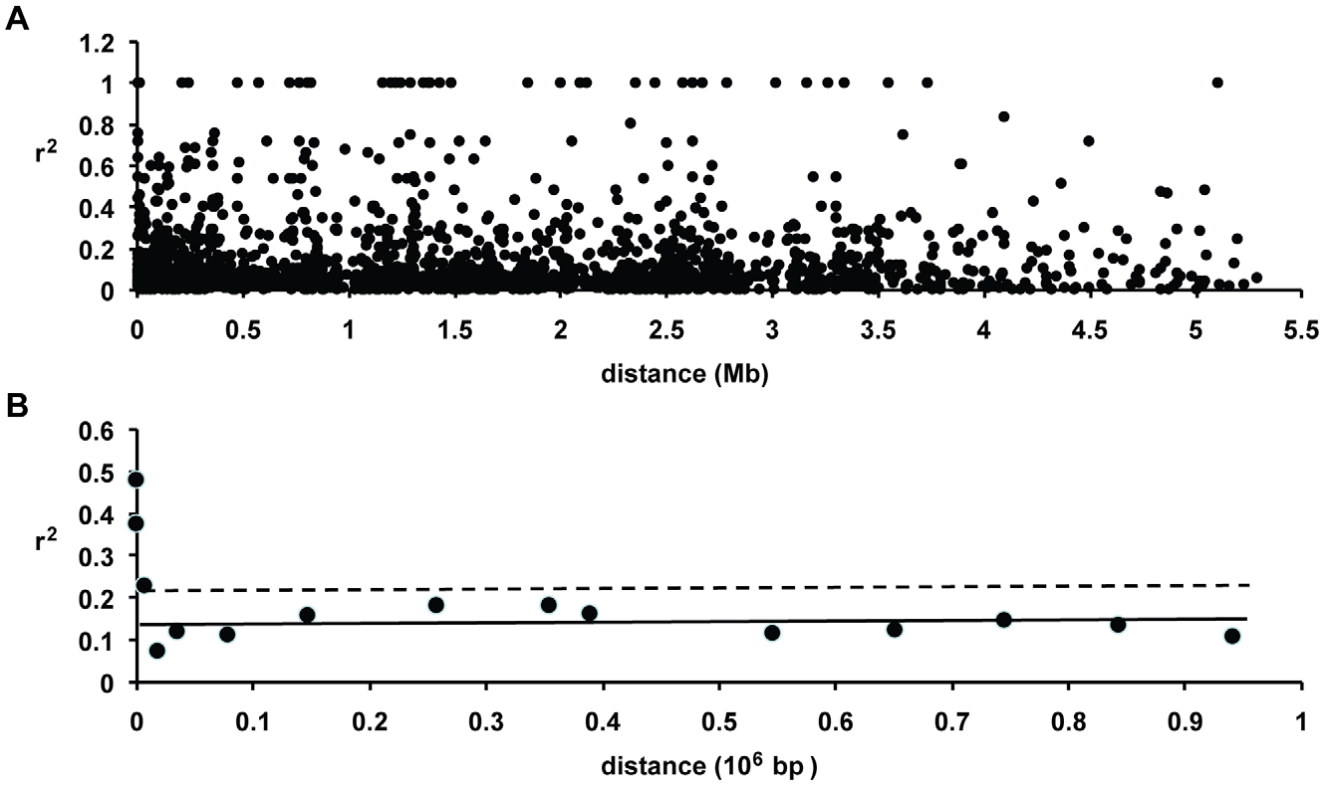
Decay of linkage disequilibrium with distance in chromosome 4. (A) Pairwise r^2^ plotted against distance. Linkage disequilibrium is calculated only for the SNPs that have at least 10% frequency in the sample. A permutation analysis indicates that the decay of LD with distance is significant (P<0.031). (B) Plot of mean r^2^ in various distance bins. The solid horizontal line gives the mean r^2^ and the dashed line is the 80^th^ percentile for unlinked markers. The baseline linkage disequilibrium in *D. discoideum* is achieved between ∼10–25 kb.

We found little evidence of high levels of linkage disequilibrium among segregating sites across this *D. discoideum* chromosome (see Figure 7). A plot of linkage disequilibrium with physical distance across chromosome 4 indicates that LD decays substantially at short distances (see Figure 8). The baseline level of LD, measured as the mean r^2^ for SNPs on separate chromosomes, is 0.136 and the 80th percentile is 0.209. Linkage disequilibrium decays to twice the baseline at less than 10 kb, and reaches the baseline level (as well as the 80th percentile) at between 10 and 25 kb (see Figure 8). More than one-fifth of the SNP pairs that were perfectly correlated (r^2^ = 1) are found within gene fragments at distances less than 300 bps, although high r^2^ values are observed even between SNPs separated by ∼5 Mb. Using a permutation test, we found that the decay in r^2^ with distance was significant (P<0.03). This test of r^2^ with distance was significant regardless of whether we included or excluded low frequency variants.

The other five chromosomes had too few SNPs in our study to obtain a meaningful estimate of LD decay with distance for each chromosome separately. When we combined all the within-chromosome data from these five other chromosomes, however, we observed a similar pattern of short-distance LD decay (see Figure S5). The LD decay over short distances on chromosome 4 is also observed if we consider only the Virginia versus geographically widespread sample, except that the latter had higher levels of LD with a baseline r^2^ of 0.182.

**Figure 9:**
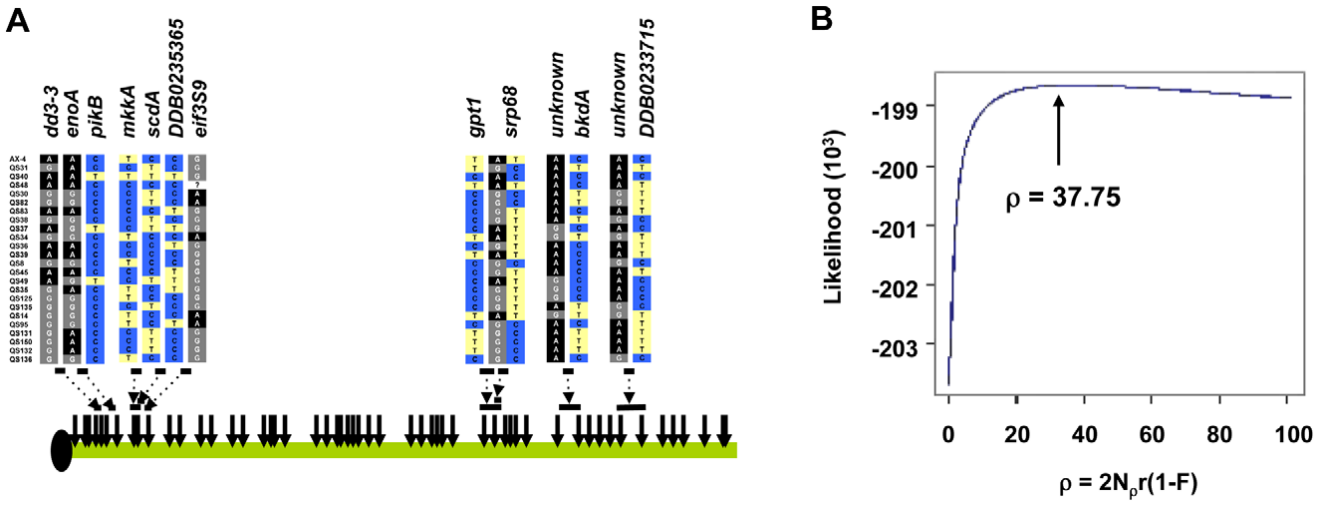
Recombination in the *D. discoideum* genome. (A) The locations of several recombination events between SNPs are indicated by the horizontal bars and the sequence at those SNPs are shown. The SNPs and the genes they are found in are shown and color-coded. Each row of SNPs represents an individual *D. discoideum* strain. The dotted arrows indicate the SNPs flanking the recombination events, which are inferred by the presence of all 4 possible genotypes between SNP pairs. (B) Composite likelihood plot of estimates of ρ. The plot shows a steep decrease in likelihood at approximately ρ<10, and the maximum is at ρ = 37.75. The likelihood surface is nearly flat at approximately ρ>20.

Application of the 4-gamete test provided direct evidence of recombination in the *Dictyostelium* genome. We observed a minimum number of recombinants (R_M_) of 14 across chromosome 4 in the 25 strains (see Figure 9). Most of these recombination events occur between gene fragments, although at least one intragenic recombination was observed. Further insight into the history of recombination in *Dictyostelium* can be gleaned from the population recombination parameter ρ, a compound parameter equal to 2N_ρ_r(1−F), where N_ρ_ is the effective population size estimated from recombinational diversity, r is the recombination rate and F is the inbreeding coefficient. Based on the SNP data across chromosome 4, we obtained a moments-based estimate of ρ = 82.46 using the method described by Wakeley [25]. To obtain a more precise estimate of ρ, however, we used an extension of the parametric method of Hudson [26] that is robust to different mutation models. The likelihood plot for the estimate shows a steep increase in likelihood from 0 to ∼20, with a maximum likelihood estimate for ρ at 37.75 (see Figure 9). A likelihood permutation test indicates that this estimate is significantly different from zero (P = 0.000); this test was significant whether we included or excluded low frequency variants. While the spacing of our markers may cause us to underestimate ρ across chromosome 4 using this methodology, it is clear from our analysis that sexual (meiotic) recombination has occurred frequently in the history of the chromosomes surveyed.

## Discussion

*D. discoideum* has one of the lowest levels of nucleotide variation observed in any eukaryotic species (see Figure 3), which indicates that very few mutations differentiate even genetically distinct strains. The estimated levels of nucleotide variation suggest that, on average, two *D. discoideum* strains should differ by only ∼27,000 SNPs over the length of its 34 Mb genome. The source of this low level of variation is unclear, but could be attributed to a population at equilibrium with small effective size, a recent bottleneck event or a low mutation rate. Little is known about the mutation rate in this species, although a low rate has been observed at microsatellite loci in mutation accumulation experiments [27].

*D. discoideum* strains do not appear to form geographically distinct subgroups in the eastern United States. There is no clear pattern of geographic structuring of nucleotide variation and no evidence of isolation-by-distance in our sample set. The formation of the fruiting body is believed to facilitate dispersal of spores [28], and these social amoebae are thought to be moved by water, small arthropods, nematodes and birds [29]–[32]. Our results suggest that these vectors lead to a high rate of dispersal, which may also explain the relative diversity in genotypes found within small population patches (e.g. the Virginia population, also see refs. 13 and 14). We should note that although we do not observe any large-scale geographic pattern among the strains, a separate study sampling from several localities has shown that there nevertheless is differentiation between *D. discoideum* populations [33].

The transition from solitary cells to a multicellular fruiting body is one of the quintessential examples of social cooperation, and the death of stalk cells for the survival of spores provides an example of altruistic behavior [7]–[12]. Although social cooperation in *D. discoideum* strains has been largely studied in cells with the same genetic background, clones of different genetic backgrounds are known to co-occur in the field [13]. It has been demonstrated, for example, that cheating behavior is commonly seen in some chimeric fruiting bodies comprised of disparate strains, in which selfish individuals (cheaters) preferentially occupy a larger proportion of spores. Moreover, kin discrimination between distinct genotypes is positively correlated with genetic distance between strains [15]. Genetic relatedness is thus a key component of the dynamics of social interaction.

We find that many of our *D. discoideum* strain pairs show evidence of kin discrimination between strains that results in assortative association in the sorus during fruiting body formation, as well as dominance of one strain over another. This indicates that social dynamics in *D. discoideum* may differ genetically between strain pairs. Our results, however, also suggest that there is no correlation between the observed SNP divergence and kin discrimination between strains in the fruiting body.

This result differs from a relatedness-based prediction of kin selection theory [1] as well as previous work that suggests kin discrimination in this species [15]. Several reasons might explain this discrepancy. First, the range of SNP differences between strains in our sample is small, and may therefore lead to poor resolution of genomic differentiation between strains. This is a direct consequence, in part, of the very low variation in this species, which limits the number of SNPs in our analysis. We obviate this somewhat by partitioning our data into those strain pairs that had low- or high-divergence with respect to the median pairwise SNP divergence in our sample. The pattern of no correlation in measures of kin discrimination with SNP divergence still holds in this analysis (see Table S1).

Second, previous studies [15] examined chimera formation with one fixed axenic line and employed highly polymorphic microsatellite loci to estimate genetic distances between pairs. Genome-wide SNP differences may not be a relevant measure of genetic relatedness in *D. discoideum* during social interactions; it is possible that what is relevant are the genetic states of specific set of genes responsible for certain social behavior, and this may differ from overall genome-wide genetic relatedness. An extreme scenario invokes greenbeard genes, which leads to cooperation specifically directed towards other individuals carrying the same allele at a particular locus [1], [34], [35]. Notably, it also appears that polymorphic *lagB1* and *lagC1* (now called *tgrB1* and *tgrC1*) genes, which encode transmembrane proteins that participate in cell adhesion and signaling, are associated with kin discrimination in *D. discoideum* [16]. This observation suggests the possibility that only a small group of genes condition social interaction during chimeric fruiting body formation.

The possibility that variation at specific loci is responsible for determining self/no-self recognition in *D. discoideum* is buttressed by our results that there is an effect of geographic distance between strain origins on kin discrimination. Our observation of greater kin discrimination among strains within a site versus between sites suggests the evolution of mechanisms to prevent chimera formation among strains that co-occur in a particular area. This is consistent with very high levels of relatedness (from 0.86 to 0.98) found among fruiting bodies in the wild [14]. If our result of greater kin discrimination in sympatry is true, then a consequence would be that strains that encounter each other in local populations preferentially form fruiting bodies with clonemates, and that this preference is diminished if strains do not encounter each other because of allopatry. Kin discrimination may thus evolve between *D. discoideum* strains in close proximity to maintain multicellular cooperation within fruiting bodies and control against invasion by cheater mutants in the wild [14].

The specific patterns of both the social behavior and population structure of *D. discoideum* may also contribute to this geographic effect on kin discrimination. Contrast our results with recent work, for example, in the social bacterium *Myxococcus xanthus*, which displays greater antagonisms among strains from different locations versus those from short spatial scales [36]. Fitness antagonisms are also observed between local strains in *M. xanthus*, but divergence among allopatric strains appears to reinforce the ability to discriminate between self and non-self [36]. This bacterium, however, appears to have strong geographic structuring of genetic variation; in comparison, our *D. discoideum* samples are not strongly structured, with a low F_st_ between Virginia versus non-Virginia samples, and the observation that genetically divergent strains co-exist in one site (see Figure 6). Moreover, *M. xanthus* exists as swarms during their predatory phase, which results in social cohesion even before fruiting body formation in this species. In contrast, *D. discoideum* cells have a distinct predatory single-cell stage, which increases the likelihood of chimera formation during periods of starvation. Both these factors – the potential presence of highly divergent strains in a given geographic area coupled with the higher probability of chimera formation – may select for stronger self/non-self recognition mechanisms for sympatric strains in *D. discoideum*.

**Figure 10:**
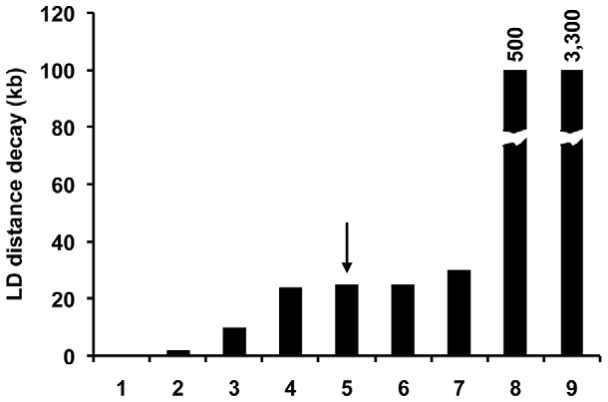
Comparison of LD distance decay between various species. For species in which the LD distance decay is reported as a range, we plotted the maximum distance. The species are 1 - *D. melanogaster*, 2 - *C. remanei*, 3 - *A. thaliana*, 4 – *S. cerevisiae*, 5 – *D. discoideum*, 6 – *S. paradoxus*, 7 – *H. sapiens*, 8 - *O. sativa*, 9 – *C. elegans*. For both *O. sativa* and *C. elegans*, the reported LD distance decay is off the scale, and the actual numbers are indicated on top of the bars. An arrow highlights the *D. discoideum* estimate. Data for this slide from other species are from previous work [21], [22], [54]–[59].

The sexual phase of *D. discoideum* also represents another distinct but poorly understood mechanism for social cooperation in this species. Unlike fruiting bodies, which are readily induced in the laboratory, the sexual phase (see Figure 1) has been difficult to study. The extent of sex in the life cycle of *D. discoideum* in nature is unclear, but molecular population genetic analysis provides an independent indicator of the importance of the sexual phase in this social species. The rapid decay of LD, the presence of recombinant strains, and the high levels of the population recombination parameter across chromosome 4 all suggest that recombination is widespread within *D. discoideum*. The pattern of LD decay is in line with those observed in other sexual species (see Figure 10). The only laboratory estimate of recombination in *D. discoideum* is from one study, which reported a high level of recombination (r = 0.001 morgans kb^−1^) [20]. If this high estimate is correct and representative of genome-wide recombination rates, we would intuitively expect a more rapid decay of LD with distance than what we observe, of the same order of magnitude as outcrossing species such as *D. melanogaster* or *C. remanei*. Our observed pattern of LD decay in *D. discoideum* suggests either that this previous estimate of recombination rate is too high, or possibly that there is a high level of inbreeding in this species.

Our results suggest that sex as a form of social interaction may be an important aspect of this organism's life cycle. Given that we find different mating types within a local population (e.g. the Mountain Lake Biological Station population in Virginia), there clearly is ample opportunity for strains to form macrocysts in nature, which support our finding of a history of recombination in the *D. discoideum* genome. One intriguing aspect of the sexual phase is that the developing zygote, upon cell fusion, signals and attracts hundreds of neighboring solitary cells and proceeds to consume them for nutrition in the process of macrocyst formation [17]–[19]. The same cAMP signal that leads to fruiting body formation appears to play a similar role in the attraction and cannibalistic consumption of free-living cells by the developing zygote, and this may represent a different mode of social cooperation. A social evolution perspective would probably consider the cannibalized cells to be altruistic rather than pure victims since they construct a cellulose prison for themselves before they are eaten [37]. It is unclear, however, what ecological conditions favor the asexual fruiting body or the sexual diploid macrocyst in nature. The evidence for sexual recombination in the wild, however, demonstrates that while fruiting bodies are readily formed in laboratory conditions, the sexual macrocyst phase may be an important aspect of the biology of *D. discoideum* in the field that needs to be closely studied on order to understand the full spectrum of phenomena associated with the social biology of this species.

Our molecular population genetic data also has wide implications for the use of this species as a model system. *D. discoideum* is already a key model organism, with an extensive community of researchers, a whole-genome sequence, an active stock center and community database. It has already provided key insights into the nature of development and differentiation, the mechanisms of cell-cell signaling, the evolution of social cooperation and the rise of multicellularity. Nevertheless, this species has yet to be used as a genetic model organism because sex has been observed infrequently in the laboratory that has hampered mutant analysis.

Our results open the possibility that *Dictyostelium* genetic studies using controlled matings may be possible once we fully understand how to bring sex and recombination from the wild into the laboratory environment. Moreover, our study of the patterns of molecular diversity and linkage disequilibrium can aid workers in designing association genetic approaches to identify genes involved in natural variation (including social interaction). These approaches can expand the potential scope of *Dictyostelium* genetic studies, and allows researchers to harness this unique and fascinating social microbe for fundamental studies in development and evolution.

## Materials and Methods

### Samples and sequencing

A panel of 24 *D. discoideum* wild strains was chosen to represent the diversity found within the species in North America (see Table S2 and Figure 2). Thirteen of these strains were collected at a single site at Mountain Lake Biological Station, Virginia while the other 11 were collected throughout the range of *D. discoideum* in North America. For our analysis, we also used the complete genome sequence from the AX-4 strain [6]. DNA was extracted from single cell cultures. Gene fragments, approximately 400–600 bp in length, from across the *Dictyostelium* genome were chosen for sequencing (see Figure 2 and Table S3). Ninety-four of these fragments were in chromosome 4, and were selected with adjacent gene fragments separated by various lengths (∼2–223 kb); this design was chosen to allow a better estimation of linkage disequilibrium. The other 44 gene fragments were chosen randomly from the other 5 chromosomes.

Primers were designed from the *D. discoideum* genomic sequence available from Dictybase (www.dictybase.org) using Primer3 [38] (see Table S4). Primers were designed in exons and flanked intron sequences within each fragment. DNA sequencing was carried out at the Cogenics sequencing facilities (New Haven, CT) as described in [39]. Base pair calls, quality score assignment and construction of contigs were carried out using the Phred and Phrap programs (Codon Code, Dedham, MA). Sequence alignment and editing were carried out with BioLign Version 2.09.1 (Tom Hall, NC State Univ.). For all analyses, the published sequence of the AX-4 strain was included [6]. From previous studies, we find that this procedure has a sequencing error rate of <0.01% [22]. All sequences are deposited in Genbank.

### Diversity and linkage disequilibrium (LD) analyses

We estimated the levels of nucleotide variation (θ_W_) [40], nucleotide diversity (π) [41] and Tajima's D [23], and F_st_ [42] across all STS fragments, and determined the frequency distributions of SNPs across the genome [43].

For the analysis of LD, only biallelic SNPs of at least 10% frequency were considered, as rare alleles can have large variances in LD estimates. We calculated the LD as the correlation coefficient r^2^ between each SNP pair [44]. LD heatmaps and illustration of SNP configurations were made using the Seattle SNPs Genome Variation Server (http://gversusgs.washington.edu/GVS/). Due to the large amount of variance in the estimates of LD for any particular SNP pair, we combined SNP pairs into distance intervals to reduce the influence of outliers and to obtain a better visual description of the LD decay with distance. For the estimate of genome-wide LD using the chromosome 4 dataset, the distance classes are <0.1 kb, 0.1–0.5 kb, 0.5–10 kb, 10–25 kb, 25–50 kb, 50–100 kb, then every 100 kb until 1Mb, and 1 Mb distance windows for SNP pairs >1Mb in distance. The use of smaller intervals at short distance scales was determined in part by the observation that LD seemed to decay substantially at these distances.

We plotted the mean of r^2^ for each distance window. We consider a particular intermarker distance interval to have LD elevated above the background level if it contains more than 10 SNP pairs and the interval mean r^2^ exceeds the 80th percentile of the unlinked pairs (which we chose from the r^2^ values between all unlinked pairwise SNPs between chromosomes). The significance of r^2^ decay with distance was tested using a permutation test with 1,000 permutations of distance with r^2^ in the data [26].

### Population stratification analyses

Population structure among *D. discoideum* strains was evaluated with STRUCTURE 2.1 using an admixture model with linkage [45]. All analyses had a burn-in length of 200,000 iterations and a run length of 200,000 iterations. Ten replicates at each value of K (population number) were carried out. Simulations were run with a model of linkage and uncorrelated allele frequencies. The appropriate K value was determined using the method of Evanno *et al.* 2005 [46] (see Figure S2). To further assess relationships among strains, all STS fragment alignments were concatenated to form a single dataset. Clustering relationships of strains based on genetic similarity were estimated with a neighbor-joining analysis as implemented in PHYLIP 3.68 [47], with distances calculated using the Kimura 2-parameter model [48]. Branch bootstrap estimates were obtained from 1000 replicates and visualization of the consensus tree was realized in FigTree v1.2.2 (http://tree.bio.ed.ac.uk/software/figtree/). Finally, based on the complete SNP dataset multiple correspondence analysis (MCA) was also carried out to investigate the pattern of population relationships using R package FactoMineR [24].

### Recombination analysis

A composite likelihood method [26] as implemented in the LDhat software was used to estimate the population recombination parameter ρ for chromosome 4. For the analysis of LD, only biallelic SNPs of at least 10% frequency were considered, as rare alleles can have large variances in LD estimates and inference of recombination has been found to be sensitive to the frequencies of alleles included in the analysis [26]. This method can also estimate recombination across the chromosome, and a likelihood permutation test with 1,000 permutations of the data was performed to test whether this recombination rate was significantly higher than zero. We used the 4-gamete test to identify recombination breakpoint intervals and estimate the minimum number of recombination events [49].

### Social assays among chimeric fruiting bodies

Twelve strain pairs that are diverged at various SNP levels were chosen for social mixing experiments [15], to test for kin discrimination and dominance (see Table S5). All strains were first grown on SM-agar plates to the mid- exponential phase, with cell densities measured by a hematocytometer. Solitary cells from each strain were then harvested, washed, and resuspended to a density of 6×10^7^ cells/ml in KK2 buffer. Two strains were mixed at 50∶50 proportions and approximately 1.5×10^7^ cells were deposited on a nitrocellulose filter. These cells were allowed to develop into fruiting bodies in a dark, humid environment at 21°C for 24 hours. The development conditions were as described [15], and a minimum of three independent cell mixing experiments was performed for each pair.

To quantify the relative abundance of individuals of each strain in chimeric mixtures, genomic DNA was extracted before and after fruiting body formation as described [15], and used for allele quantification by pyrosequencing [50]. Two SNP sites with distinct polymorphisms in separate genes from the two strains in each pair were used as genetic markers to differentiate individuals. Although 50∶50 of each strain was the targeted ratio, we expected deviations from this proportion; to control for experimental variation in mixture proportions, genomic DNA was extracted from 20 uL of cell mixtures before plating to determine the actual proportions in the input cell mixtures. Sixteen fruiting bodies from each of the three replicate plates were also picked and DNA extracted from each fruiting body.

SNP pyrosequencing primers were designed by PSQ Assay Design Software V1.0.6 and pyrosequencing reactions were carried out on PSQ 96MA (Biotage, Stockholm, Sweden). To construct standard curves for each SNP, genomic DNA from strains carrying distinct alleles at the marker SNP sites were mixed at specific proportions ranging from 0 to 1 for a final concentration of 10 ng/ul (see Figure S6). Three independent mixtures were made at each proportion to serve as replicates. Each replicate was PCR amplified and pyrosequencing carried out separately as described [50]. Peak heights measured from the three replicates were plotted against known allele proportions, and a least-squares linear regression model was used to estimate relative levels of alleles in the experiment. The two alternate SNPs serve as technical replicates, and were measured on the same genomic DNA from each fruiting body.

We developed a metric for the dominance status of one strain over a second in a chimeric mixing experiment. We define dominance as a general term to mean the difference in proportion of a strain in the fruiting body relative to the starting proportions in the single cell mixtures. The dominance value *d* is given by
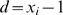
where
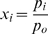
and *p_i_* represents the frequency of one strain in a fruiting bod*y*, while *p_o_* represents the frequency of the same strain in the initial cell mix.

Discrimination manifests itself when strains preferentially form fruiting bodies with genetically identical cells (clonemates). To examine kin discrimination in the formation of chimeric fruiting bodies, we employed three approaches. We estimated the relatedness (*r*) [1] within fruiting bodies relative to other fruiting bodies resulting from the cell mixture [51]. If a strain has frequency *p_i_* in the i^th^ fruiting body, and the average over fruiting bodies in the sample is *p*, its relatedness in that fruiting body can be estimated as
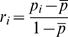



This measure, *r_i_*, is the observed deviation in frequency of a strain relative to the mean frequency, scaled by the maximum possible deviation. For the other strain, the frequency in the cell mix is 1−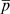 and in the i^th^ fruiting body is 1−*p_i_*, so relatedness for this strain is
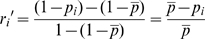
We sum over replicate fruiting bodies to obtain a measure of global change in relatedness for a pair of strains [51]. In each fruiting body, there is a fraction, *p_i_*, of individuals that have a relative change in relatedness, *r_i_*, and 1−*p_i_* individuals with r_i_′. Summing over all fruiting bodies and including both strains we obtain a global relatedness
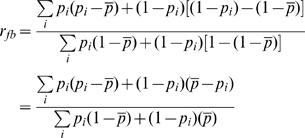
that represents how strongly strains are clustered in different fruiting bodies. It can range from zero for random clustering to one for complete segregation.

We also used two alternate measures to infer the extent of kin discrimination. Levene's statistic (*LS*) [52] is a measure of individual deviation from the mean, and is given as
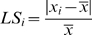
The mean of Levene's statistic was calculated for dominant strains in each mixing experiment and reduces any possible confounding effects of the variance with the mean. Finally, we estimated the variance of the arcsine square-root transform of *x_j_* and utilized it as a measure of kin discrimination as described in a previous study of *D. discoideum* [15].

### Mating type assay

Mating type tests were carried out as previously described [53]. Tests were performed in 0.5 ml LP agar, where 0.5 ml Bonner's salt solution and 5 µl *K. aerogenes* were loaded before spores from *D. dictyostelium* strains were added. Either NC4 (mating type A1) or V12 (mating type A2) was used as standard strain in a single test. Every strain of interest was assessed versus NC4, V12 and itself with three replicates each. Twenty-four well plates containing strains being tested were wrapped in aluminum foil and incubated for seven days at 21°C without shaking. Examination for formation of macrocysts was performed under 10× magnification. If we observed macrocysts in two or more replicates, the reaction was considered to be positive. If macrocysts formed with only V12, the mating type of the strain was matA1. If macrocysts formed with only NC4, the mating type of the strain was matA2. If macrocysts formed with both, the strain was ambivalent, and designated as matA3.

## Supporting Information

Figure S1 Population structure analysis. (A) Multiple Correspondence Analysis of SNP variation among *D. discoideum* strains. The locations of the strains in MCA space are indicated and show clustering among similar strains. The first three principal components are the axes of the plot and axes 1 through 3 explain 16%, 14.16%, and 12.76% of the variation. (B) Population ancestry of strains using the Bayesian program STRUCTURE. The colors give the relative proportions of the strain genomes that are attributable to a particular cluster in a structure run with K = 6.
(3.00 MB TIF)

Figure S2 Likelihood plots of population stratification analysis among *D. discoideum* strains. The likelihoods (left) and second order rate of change in likelihoods (right) of different K values are shown [46]. The likelihood begins to plateau at K = 6 and a maximum second order rate of change at K = 8.
(3.00 MB TIF)

Figure S3 Plot of Levene's statistic versus SNP divergence between strain pair. Box-plots for the two SNP markers and three replicates for each SNP marker are shown, so each strain pair is represented by six box-plots. All the box-plots are arranged according to increasing pair-wise SNP differences between strains. The vertical line gives the upper and lower limits, while the boxes indicate the upper and lower quantiles.
(3.00 MB TIF)

Figure S4 Relationship between the variance in strain proportion (arcsin square-root transformed) and pairwise SNP differences between strains. The two estimates at a given divergence level is based on the separate pairwise strain, while the standard error is calculated from the three replicates for each experiment.
(3.00 MB TIF)

Figure S5 Linkage disequilibrium decay with distance among *D. discoideum* strains, based on data across all other chromosomes except chromosome 4. The baseline linkage disequilibrium in *D. discoideum* is achieved between ∼10–25 kb. The point at 1.2 Mb is for all distance classes >1 Mb. The high LD at ∼0.6 Mb is an outlier, due to having three datapoints in that distance class with two SNP pairs in perfect LD.
(3.00 MB TIF)

Figure S6 Examples of two pyrosequencing standard curves for relative SNP proportions in *D. discoideum* DNA. The triangle, circle and square are for three different replicate mixtures at each proportion. The r^2^ values for these standard curves are 0.998 (left) and 0.999 (right). For SNPs that are found in sites with consecutive identical nucleotides, the intercept and/or slope will differ.
(3.00 MB TIF)

Table S1 Social parameters in low- and high-divergence strain pairs.
(0.03 MB DOC)

Table S2 Strains.
(0.05 MB DOC)

Table S3 Location of genes/gene fragments sequenced.
(0.15 MB DOC)

Table S4 PCR Primers.
(0.11 MB XLS)

Table S5 Strain pairs used in chimera mixing experiments.
(0.03 MB DOC)
