# Supplementary figures and images for: Variation, Sex, and Social Cooperation: Molecular Population Genetics of the Social Amoeba Dictyostelium discoideum
Source: PLoS Genet. 2010 Jul 1;6(7):e1001013. doi: 10.1371/journal.pgen.1001013 (PMC2895654; doi:10.1371/journal.pgen.1001013)

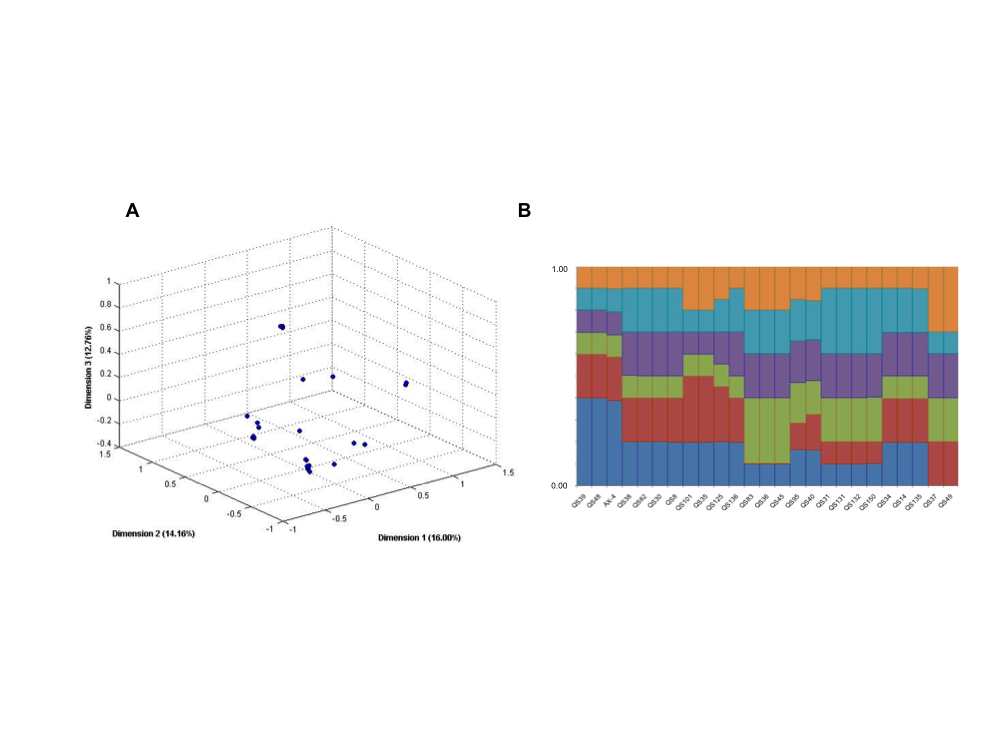

Supplement: Figure S1 — Population structure analysis. (A) Multiple Correspondence Analysis of SNP variation among D. discoideum strains. The locations of the strains in MCA space are indicated and show clustering among similar strains. The first three principal components are the axes of the plot and axes 1 through 3 explain 16%, 14.16%, and 12.76% of the variation. (B) Population ancestry of strains using the Bayesian program STRUCTURE. The colors give the relative proportions of the strain genomes that are attributable to a particular cluster in a structure run with K = 6. (3.00 MB TIF) [file pgen.1001013.s001.tif]

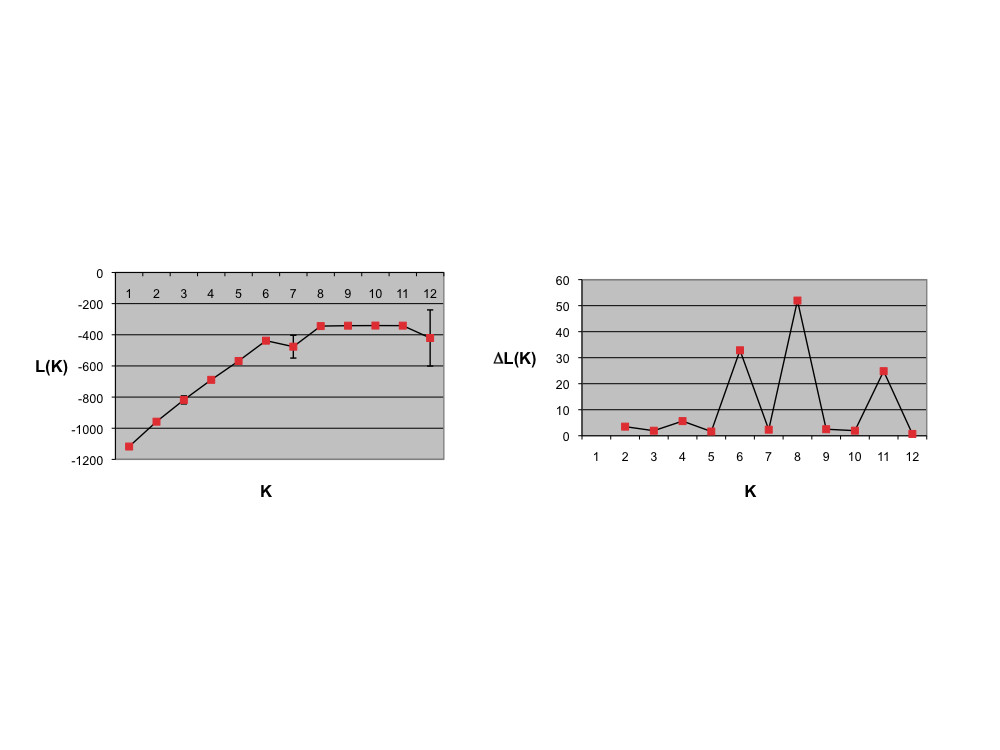

Supplement: Figure S2 — Likelihood plots of population stratification analysis among D. discoideum strains. The likelihoods (left) and second order rate of change in likelihoods (right) of different K values are shown [46]. The likelihood begins to plateau at K = 6 and a maximum second order rate of change at K = 8. (3.00 MB TIF) [file pgen.1001013.s002.tif]

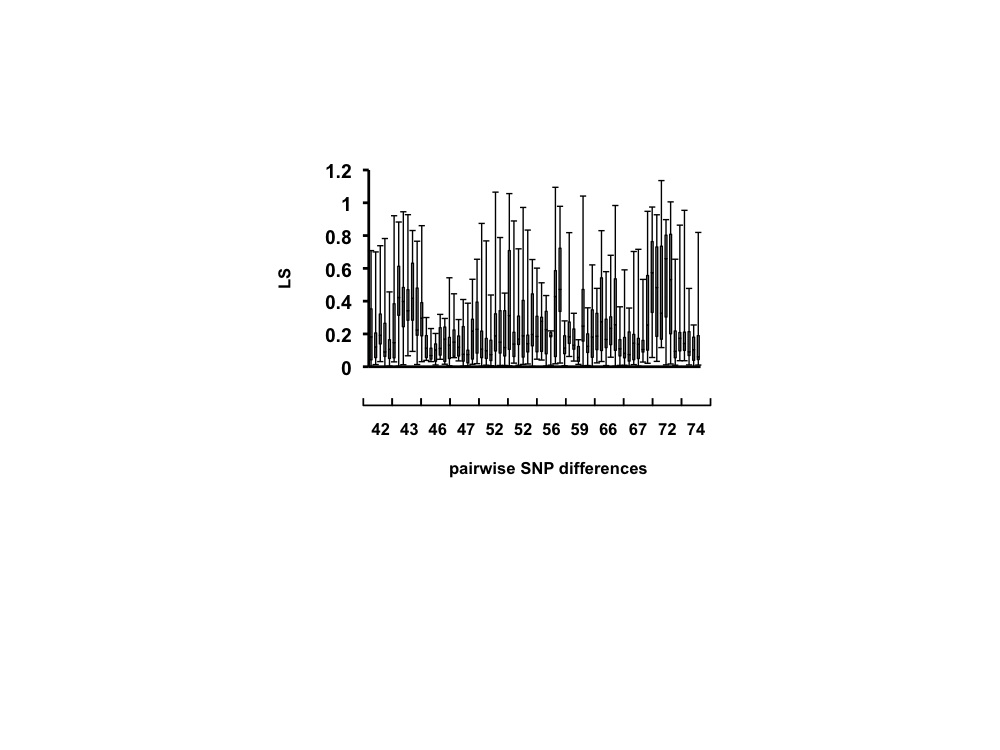

Supplement: Figure S3 — Plot of Levene's statistic versus SNP divergence between strain pair. Box-plots for the two SNP markers and three replicates for each SNP marker are shown, so each strain pair is represented by six box-plots. All the box-plots are arranged according to increasing pair-wise SNP differences between strains. The vertical line gives the upper and lower limits, while the boxes indicate the upper and lower quantiles. (3.00 MB TIF) [file pgen.1001013.s003.tif]

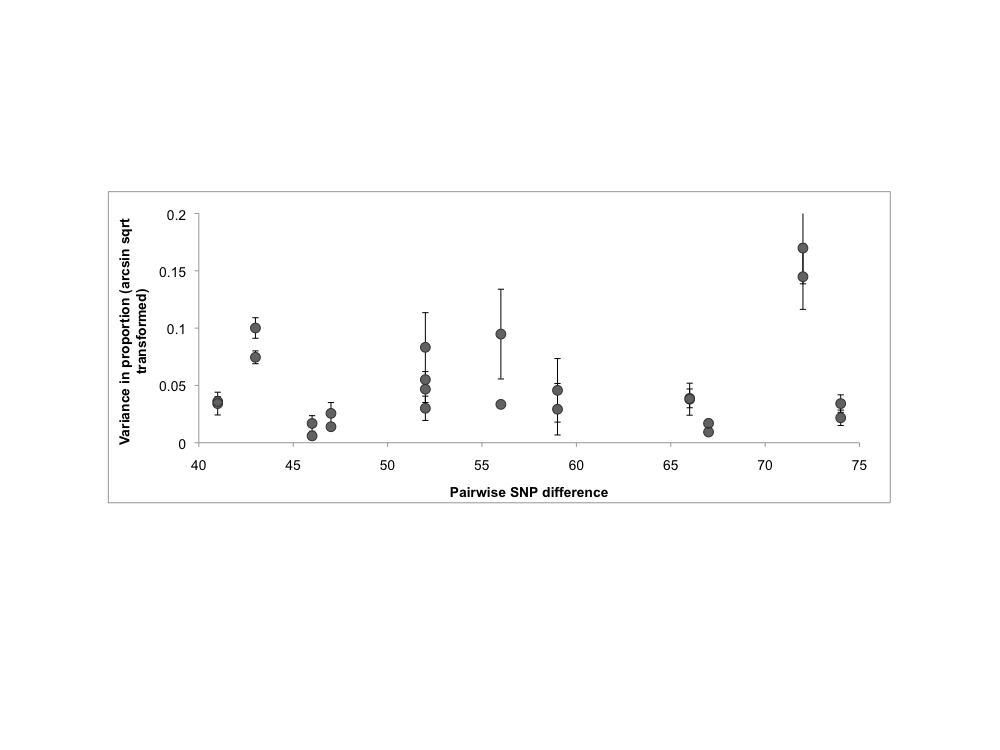

Supplement: Figure S4 — Relationship between the variance in strain proportion (arcsin square-root transformed) and pairwise SNP differences between strains. The two estimates at a given divergence level is based on the separate pairwise strain, while the standard error is calculated from the three replicates for each experiment. (3.00 MB TIF) [file pgen.1001013.s004.tif]

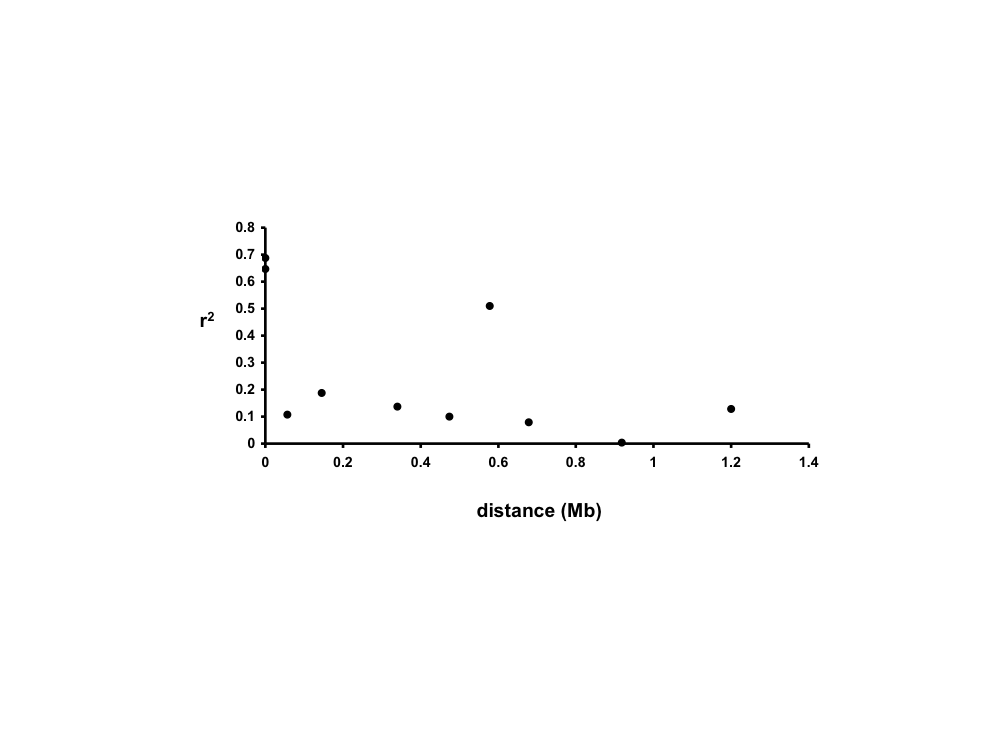

Supplement: Figure S5 — Linkage disequilibrium decay with distance among D. discoideum strains, based on data across all other chromosomes except chromosome 4. The baseline linkage disequilibrium in D. discoideum is achieved between ∼10–25 kb. The point at 1.2 Mb is for all distance classes >1 Mb. The high LD at ∼0.6 Mb is an outlier, due to having three datapoints in that distance class with two SNP pairs in perfect LD. (3.00 MB TIF) [file pgen.1001013.s005.tif]

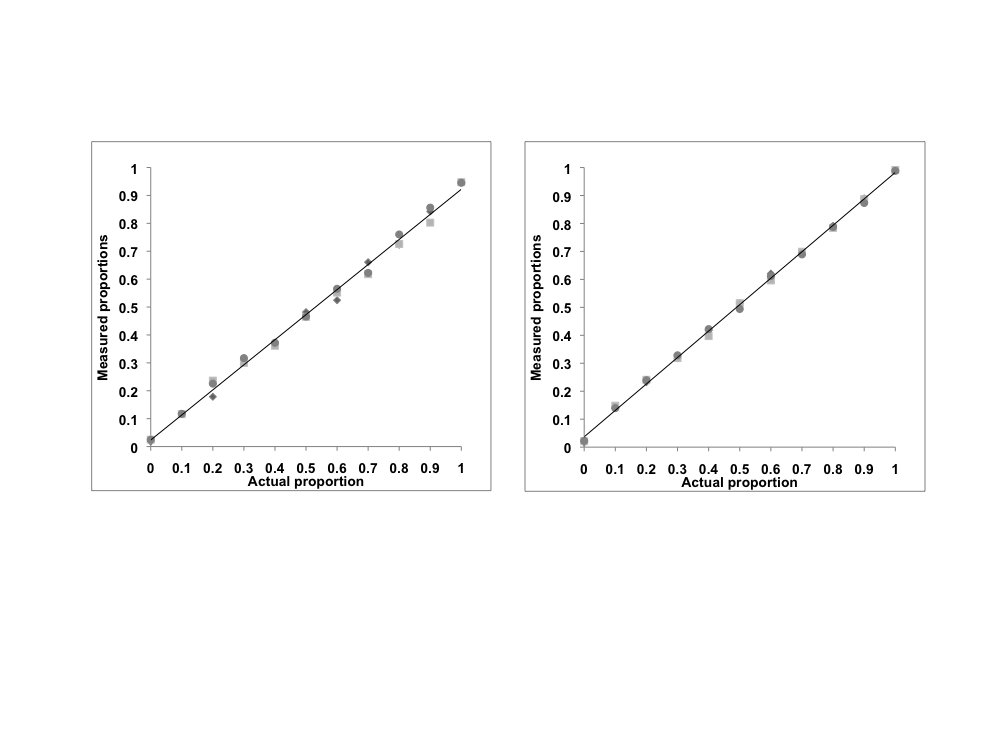

Supplement: Figure S6 — Examples of two pyrosequencing standard curves for relative SNP proportions in D. discoideum DNA. The triangle, circle and square are for three different replicate mixtures at each proportion. The r2 values for these standard curves are 0.998 (left) and 0.999 (right). For SNPs that are found in sites with consecutive identical nucleotides, the intercept and/or slope will differ. (3.00 MB TIF) [file pgen.1001013.s006.tif]
