# Supplementary material for: Variation, Sex, and Social Cooperation: Molecular Population Genetics of the Social Amoeba Dictyostelium discoideum
Source: PLoS Genet. 2010 Jul 1;6(7):e1001013. doi: 10.1371/journal.pgen.1001013 (PMC2895654; doi:10.1371/journal.pgen.1001013)
Supplement: Table S1 — Social parameters in low- and high-divergence strain pairs. (0.03 MB DOC) [file pgen.1001013.s007.doc]

| **parameter** | **low** | **high** |
| --- | --- | --- |
| *r_fb_* | 0.129 ± 0.069 | 0.181 ± 0.049 |
| *LS* | 0.226 ± 0.057 | 0.262 ± 0.049 |
| *Var[Tr(data)]^a^* | 0.038 ± 0.017 | 0.056 ± 0.021 |
| *d* | 0.075 ± 0.017 | 0.073 ± 0.023 |

^a^mean variance of the arcsin square-root transformation of the

strain proportion in the fruiting bodies, corrected for input cell

mixture.
