# Supplementary material for: Variation, Sex, and Social Cooperation: Molecular Population Genetics of the Social Amoeba Dictyostelium discoideum
Source: PLoS Genet. 2010 Jul 1;6(7):e1001013. doi: 10.1371/journal.pgen.1001013 (PMC2895654; doi:10.1371/journal.pgen.1001013)
Supplement: Table S2 — Strains. (0.05 MB DOC) [file pgen.1001013.s008.doc]

| **QS NUMBER** | **OLD STRAIN DESIGNATION** | **LOCATION** |
| --- | --- | --- |
| QS101 | S109 | Arkansas-Forest City |
| QS125 | V319A | Virginia- Mountain Lake Biological Station |
| QS131 | V331C2 | Virginia- Mountain Lake Biological Station |
| QS132 | V337D1 | Virginia- Mountain Lake Biological Station |
| QS135 | V323D1 | Virginia- Mountain Lake Biological Station |
| QS136 | V342A2 | Virginia- Mountain Lake Biological Station |
| QS14 | V326D1 | Virginia- Mountain Lake Biological Station |
| QS150 | V335B1 | Virginia- Mountain Lake Biological Station |
| QS30 | S202 | Texas- Carthage |
| QS31 | HD37D1 | Texas- Houston Arboretum |
| QS34 | S71 | Indiana- Bloomington (Lobelia) |
| QS35 | V316A1 | Virginia- Mountain Lake Biological Station |
| QS36 | S72 | Kentucky- Land Between the Lakes |
| QS37 | S67 | Texas- Linden |
| QS38 | S30 | Virginia- Mountain Lake Biological Station |
| QS39 | TN39F2 | Tennessee- Indian Gap |
| QS40 | MA12C1 | Massachusetts- Mt. Greylock |
| QS45 | V303A2B | Virginia- Mountain Lake Biological Station |
| QS48 | NC28C1 | North Carolina- Linville Falls |
| QS49 | V305B2 | Virginia- Mountain Lake Biological Station |
| QS8 | V301B1 | Virginia- Mountain Lake Biological Station |
| QS82 | S203 | Illinois- Effingham |
| QS83 | S221 | Missouri- St. Louis |
| QS95 | V330B1 | Virginia- Mountain Lake Biological Station |
