# Supplementary material for: Variation, Sex, and Social Cooperation: Molecular Population Genetics of the Social Amoeba Dictyostelium discoideum
Source: PLoS Genet. 2010 Jul 1;6(7):e1001013. doi: 10.1371/journal.pgen.1001013 (PMC2895654; doi:10.1371/journal.pgen.1001013)
Supplement: Table S3 — Location of genes/gene fragments sequenced. (0.15 MB DOC) [file pgen.1001013.s009.doc]

| **chromosome** | **gene** | **start** | **end** |
| --- | --- | --- | --- |
| 1 | JC1V2_0_00060 | 109209 | 108706 |
| 1 | SONA | 1169436 | 1168935 |
| 1 | POLA | 1470098 | 1469539 |
| 1 | JC1V2_0_00596 | 1482177 | 1482723 |
| 1 | JC1V2_0_00609 | 1512878 | 1513372 |
| 1 | JC1V2_0_00735 | 1847425 | 1846934 |
| 1 | JC1V2_0_00932 | 2304851 | 2305382 |
| 1 | GDT6 | 2914125 | 2914706 |
| 1 | DCSA | 3501237 | 3500762 |
| 1 | DCD1A | 4599648 | 4600139 |
| 2 | GENEDDB0233292 | 908732 | 909221 |
| 2 | GENEDDB0231693 | 2698677 | 2699156 |
| 2 | JC2V2_0_01116 | 2784310 | 2783834 |
| 2 | JC2V2_0_01333 | 3361385 | 3361928 |
| 2 | GENEDDB0233714 | 4220620 | 4220123 |
| 2 | JC2V2_0_01936 | 4864806 | 4865303 |
| 2 | JC2V2_0_02122 | 5365725 | 5366194 |
| 2 | JC2V2_0_02908 | 7330652 | 7330074 |
| 2 | GENEDDB0229923 | 7810895 | 7810399 |
| 2 | GENEDDB0233776 | 8010560 | 8010031 |
| 3 | JC3V2_0_00183 | 429165 | 429636 |
| 3 | SHKC | 837058 | 836538 |
| 3 | DYMB | 1082622 | 1082129 |
| 3 | PGTD | 1199706 | 1200177 |
| 3 | JC3V2_0_00796 | 2039295 | 2038776 |
| 3 | JC3V2_0_00824 | 2117715 | 2118246 |
| 3 | JC3V2_0_00872 | 2251764 | 2251276 |
| 3 | GENEDDB0231544 | 3149812 | 3150281 |
| 3 | SERA | 4008446 | 4008963 |
| 3 | JC3V2_0_02277 | 5707273 | 5707768 |
| 4 | BC4V2_0_00012 | 23729 | 23146 |
| 4 | LVSE | 97082 | 97655 |
| 4 | RAD54 | 119823 | 120331 |
| 4 | MCM8 | 150021 | 149524 |
| 4 | BC4V2_0_00071 | 176414 | 175963 |
| 4 | ORCA | 183103 | 182644 |
| 4 | BC4V2_0_00077 | 189200 | 188743 |
| 4 | DD3-3 | 211298 | 210835 |
| 4 | ENOA | 226460 | 225945 |
| 4 | PIKB | 249021 | 248501 |
| 4 | BC4V2_0_00119 | 299796 | 299346 |
| 4 | KMO | 360162 | 359637 |
| 4 | GENEDDB0238848_PS | 368930 | 368369 |
| 4 | GENEDDB0216320 | 388853 | 388310 |
| 4 | GENEDDB0216320 | 391184 | 390604 |
| 4 | BC4V2_0_00201 | 497349 | 497830 |
| 4 | BC4V2_0_00203 | 507400 | 506841 |
| 4 | GENEDDB0231374 | 512025 | 511515 |
| 4 | DCP2 | 516880 | 517382 |
| 4 | MKKA | 531357 | 531891 |
| 4 | BC4V2_0_00218 | 554108 | 554558 |
| 4 | SCDA | 574492 | 574941 |
| 4 | GENEDDB0235365 | 593904 | 593404 |
| 4 | GENEDDB0229872 | 609112 | 608644 |
| 4 | GENEDDB0238064 | 811304 | 810801 |
| 4 | EIF3S9 | 848413 | 848983 |
| 4 | CUTA | 909279 | 909772 |
| 4 | BC4V2_0_00411 | 1042404 | 1041946 |
| 4 | GRLN | 1198825 | 1198368 |
| 4 | CTSB | 1297807 | 1298305 |
| 4 | SEC1 | 1337212 | 1337669 |
| 4 | BC4V2_0_00528 | 1374302 | 1374757 |
| 4 | PIGA | 1383835 | 1384308 |
| 4 | GENEDDB0233340 | 1610741 | 1610163 |
| 4 | BC4V2_0_00625 | 1631401 | 1631972 |
| 4 | BC4V2_0_00626 | 1636824 | 1637332 |
| 4 | GENEDDB0220640 | 1647993 | 1647468 |
| 4 | BC4V2_0_00635 | 1669488 | 1670010 |
| 4 | BC4V2_0_00636 | 1672491 | 1673005 |
| 4 | PROS | 1680478 | 1681077 |
| 4 | KYNU | 1686197 | 1685615 |
| 4 | EIF2A | 1800676 | 1800090 |
| 4 | BC4V2_0_00779 | 2022705 | 2023243 |
| 4 | BC4V2_0_00782 | 2029383 | 2029965 |
| 4 | KSNH | 2105363 | 2104883 |
| 4 | BC4V2_0_00813 | 2118805 | 2118338 |
| 4 | BC4V2_0_00823 | 2146408 | 2145872 |
| 4 | GLYS | 2182443 | 2182926 |
| 4 | GENEDDB0233096 | 2216469 | 2216977 |
| 4 | D7 | 2277341 | 2276782 |
| 4 | GCSA | 2292341 | 2292816 |
| 4 | BC4V2_0_00878 | 2305284 | 2305746 |
| 4 | BC4V2_0_00897 | 2352523 | 2353019 |
| 4 | GENEDDB0230138 | 2364748 | 2364197 |
| 4 | GENEDDB0252814 | 2370381 | 2370853 |
| 4 | GENEDDB0231540 | 2379632 | 2379126 |
| 4 | BC4V2_0_00908 | 2388958 | 2389532 |
| 4 | COPB | 2395822 | 2396378 |
| 4 | BC4V2_0_00915 | 2410459 | 2409993 |
| 4 | ABCC8 | 2583342 | 2582782 |
| 4 | BC4V2_0_01073 | 2816999 | 2817515 |
| 4 | BC4V2_0_01078 | 2824806 | 2824330 |
| 4 | BC4V2_0_01086 | 2839974 | 2840442 |
| 4 | BC4V2_0_01090 | 2848338 | 2848903 |
| 4 | KSND | 2860820 | 2861328 |
| 4 | BC4V2_0_01098 | 2866691 | 2867242 |
| 4 | RAD54B | 2874408 | 2873813 |
| 4 | STT3 | 2901081 | 2901660 |
| 4 | GENEDDB0231234 | 2966059 | 2965552 |
| 4 | BC4V2_0_01144 | 2969603 | 2969142 |
| 4 | SCRA | 3056005 | 3056549 |
| 4 | GENEDDB0232423 | 3095317 | 3094734 |
| 4 | ATG9 | 3195961 | 3196540 |
| 4 | POLD1 | 3207730 | 3207245 |
| 4 | NUP107 | 3400701 | 3400229 |
| 4 | FORH | 3418141 | 3418595 |
| 4 | GENEDDB0237915 | 3495472 | 3494897 |
| 4 | BC4V2_0_01421 | 3603672 | 3604135 |
| 4 | GENEDDB0235380 | 3667772 | 3667286 |
| 4 | GENEDDB0235380 | 3672171 | 3671684 |
| 4 | GPT1 | 3693951 | 3694519 |
| 4 | SRP68 | 3702629 | 3703162 |
| 4 | GXCP | 3731537 | 3732133 |
| 4 | GENEDDB0237545 | 3753795 | 3754256 |
| 4 | GENEDDB0232139 | 3801667 | 3801215 |
| 4 | BC4V2_0_01571 | 3998671 | 3999231 |
| 4 | BC4V2_0_01648 | 4206544 | 4207137 |
| 4 | BKDA | 4251011 | 4251510 |
| 4 | ARGC | 4257696 | 4257114 |
| 4 | GENEDDB0231643 | 4296128 | 4295647 |
| 4 | U2AF2 | 4406451 | 4407015 |
| 4 | BC4V2_0_01800 | 4592720 | 4593245 |
| 4 | GENEDDB0233715 | 4792313 | 4792885 |
| 4 | VPS13A | 4897762 | 4897169 |
| 4 | BC4V2_0_01979 | 5025649 | 5026155 |
| 4 | BC4V2_0_02058 | 5230065 | 5229587 |
| 4 | GENEDDB0237550 | 5408480 | 5408024 |
| 4 | CYSS | 5413882 | 5413414 |
| 5 | GENEDDB0231138 | 48862 | 48325 |
| 5 | GENEDDB0235221 | 261457 | 260965 |
| 5 | BC5V2_0_00176 | 406981 | 407563 |
| 5 | BC5V2_0_00312 | 740164 | 739657 |
| 5 | NCFA | 2005419 | 2004831 |
| 5 | GEFAA | 3028763 | 3028233 |
| 5 | BC5V2_0_01370 | 3506576 | 3506127 |
| 5 | BC5V2_0_01523 | 3900355 | 3899763 |
| 5 | FTHS | 4032643 | 4033203 |
| 5 | GENEDDB0237460_PS | 4519603 | 4520080 |
| 6 | CCT2 | 178127 | 177608 |
| 6 | NUP98 | 240614 | 241105 |
| 6 | BEC6V2_0_00418 | 1079808 | 1080310 |
| 6 | GENEDDB0232106 | 1123079 | 1122493 |
| 6 | BEC6V2_0_00592 | 1554603 | 1554092 |
| 6 | BEC6V2_0_00630 | 1658187 | 1658757 |
| 6 | MHSP70 | 2763183 | 2763753 |
| 6 | GENEDDB0231848 | 2811725 | 2811135 |
| 6 | SF1 | 3036245 | 3036803 |
| 6 | BEC6V2_0_01249 | 3161675 | 3162175 |
| 6 | BEC6V2_0_01275 | 3231175 | 3231649 |
