# Supplementary material for: Variation, Sex, and Social Cooperation: Molecular Population Genetics of the Social Amoeba Dictyostelium discoideum
Source: PLoS Genet. 2010 Jul 1;6(7):e1001013. doi: 10.1371/journal.pgen.1001013 (PMC2895654; doi:10.1371/journal.pgen.1001013)
Supplement: Table S5 — Strain pairs used in chimera mixing experiments. (0.03 MB DOC) [file pgen.1001013.s011.doc]

| **Strain 1** | **Strain 2** | **allopatric (A)**  **or sympatric (S)** |
| --- | --- | --- |
| QS83 | QS45 | A |
| QS83 | QS37 | A |
| QS37 | QS49 | A |
| QS37 | QS131 | A |
| QS131 | QS132 | S |
| QS131 | QS83 | A |
| QS36 | QS39 | A |
| QS36 | QS49 | A |
| QS49 | QS35 | S |
| QS49 | QS150 | S |
| QS150 | QS34 | A |
| QS150 | QS36 | A |
